# The Clinical Society of Maryland

**Published:** 1893-08

**Authors:** H. O. Reik

**Affiliations:** Secretary pro tem; 810 Madison Avenue


### THE CLINICAL SOCIETY OF MARYLAND.

Baltimore, April 7th, 1893. The 279th regular meeting was called to order by the President, Dr. Wm. E. Moseley.

Dr. Samuel Theobald read a paper on radical cure of stricture of the nasal duct. He said: Fifteen years ago I called attention to the ineffectual use of small probes in the treatment of lachrymal strictures. Dr. Williams and Dr. Noyes also spoke about it at the same time.

To-day, while using larger probes than formerly, the majority of the profession are not using as laige ones as myself. Some of my first critics said that it was impracticable to insert such probes and that I must have overlooked the anatomical arrangement of the canal. The fact was that anatomical observation was the foundation of my theory. I examined a number of skulls and cadavers and I measured the ducts by seeing how large a probe I could pass. In experiments on dry skulls I found that in 70 ducts probed the average size was 4.7 mm in diameter. I have in my hand a pamphlet of recent date in which it is claimed that the probes cannot be passed even in the bony canals. As a matter of fact it is really easier to pass the large probes on the cadavers because there is more give to the tissues and you can use more force than is possible in the dry skull without breaking the bones. Of ten subjects examined I found the average size of the duct to be 4.47 mm in diameter, the largest being 7 mm and the smallest 3 mm which is equal to a No. 12 probe. Why should we use such large probes? Because the small ones will not produce permanent benefit. DeSchweinitz in his latest work ('92) speaks as if he still used Bowman's probes, but lays no stress upon the use of large ones and gives a doubtful prognosis. Fuchs in his new work ('92) does not go beyond a No. 6 and as the prognosis says: "Even in the most favorable cases, treatment lasts for many weeks and a recurrence is the rule." After an experience of 15 years with their use I can say that there is no class of cases which I approach with more confidence of a successful issue. Of course when we have to deal with a severe case of nasal catarrh or oezena, we cannot expect the most marked success. Many of the cases I see have been treated before by small probes. I have watched this treatment for years and have seen no relapses in persons who were discharged as cured. I believe nearly all the relapses in patients who have for one reason or another ceased before treatment was completed. I invariably endeavor to avoid beginning with a small probe for it is so easy to produce with them a false passage. I prefer to begin with a No. 5 or 6. There are three kinds of tissues in the duct, mucous membrane, periostium and bone. If the closure is due to simple hypertrophy of the membrane it will probably be relieved by simple collyria. I have never met with blenorrhœa of the lachrymal sac caused by severe ophthalmia. It is almost invariably due to extension of catarrh from the nose. My method of operating is first to anæsthetise with cocaine and then pass a small tube, No. 2 usually, through the puncta and canaliculus to look for. This facilitates the entrance of the probe-canaliculus knife, into the sac. Having slit up the canal I then pass a No. 5 or No. 6 probe or a smaller one if I fail with these. I anoint the probes with vaseline containing 10 per cent. cocaine. Having surely entered the sac we need not hesitate to use force in passing the stricture. I have never seen any serious consequences. Rarely I have had while using the small probes an ecchymosis of the lid and once or twice slight inflammatory reaction. I do not think it advisable to probe daily unless compelled to for want of time. It may excite too much inflammation. Every other day is my custom, I increase by one number each time, skipping a number if very freely passed or dropping back one if too tight. In two-thirds of all cases, including children, I have used No. 16. Having reached largest, I intend to use, I then increase the intervals. The only objection I know to the treatment is that the duct may remain too pervious and air passes freely when the nose is blown, but such inconvenience is, I think, very small. In addition to probing I always prescribe collyria to be used three times daily. The most useful ones I have found to be a solution of bichloride of mercury 1-12000. Next to that I prefer solution of alum, 10 grs. to the ounce. I do not attack a fistula or carious bone; they soon take care of themselves if the passage is open. Patients may be taught to probe themselves with the larger probes. Stricturotomy has never appealed to me as rational treatment nor have I ever had any reason to destroy the lachrymal sac.

Dr. Harlan said that almost 12 years ago he began to investigate Dr. Theobald's probes. Experiments on the cadaver showed him that the largest one equal to a No. 32, could be passed in most cases, and the second size in all cases without difficulty. At the Presbyterian Eye and Ear Hospital all the cases of stricture at that time were turned over to him. He used the probes and found them particularly satisfactory. He dilated many up to 16, but with not quite as good results as Dr. Theobald obtained. He had most trouble with old cases probably because the first probe had not been properly passed, and either had not entered the sac or had made a false passage or had injured the periostium. Cases in which he could hear the scraping against the bone were most unfavorable and often did not remain open after a No. 16 was used. He has made it a rule not to go beyond No. 12. He still finds some cases closing up again. Like Dr. Theobald, he begins with No. 6. He has found much satisfaction in certain cases by the use of a lead style. He reported a case which had been probed up to No. 16 and in which a style had been used but still the stricture recurred at intervals. In a second case where there was no puncta, he made one and probed to No. 10. There was recurrence after a few days. The treatment was repeated with the same result. A style was used but the result was the same. Cases where there is diseased bone, opposing raw surfaces, or stricture at the extreme lower end of the duct, he has found hard to treat. For the large majority of cases he has only words of praise for Dr. Theobald's method.

Dr. Bernstein said that before going abroad he had treated cases up to No. 12 and now after two years he finds them still perfectly well. He had taken a set of the probes abroad with him and had fairly astonished the German physicians with them.

Dr. Wicker, when slitting up the canaliculus attempts to pass the knife as far as possible to cut any stricture. Dr. Theobald considered the use of collyria in addition to probing an important point. He has rarely found any difficulty in passing the same sized probe after the lapse of a week or two. Roughness of bone does not lead him to change his prognosis. Such cases do require a longer time, but he expects satisfactory results. H. O. REIK, Secretary *pro tem.*

810 Madison Avenue.

